# Old and New Remedies

**Published:** 1876-07

**Authors:** W. F. Barr

**Affiliations:** Abingdon, Va.


THE
### SOUTHERN MEDICAL RECORD.

VOL. VI. JULY, 1876. NO. 7

### Original Communications.

### "OLD AND NEW REMEDIES."

By W. F. BARR, M. D., Abingdon, Va., (read before Abingdon Academy of Medicine. Published by request.)

I heard of a young man whose custom it was to go to mill with a bushel of corn in one end of the bag, and a large stone in the other with which to balance the bushel of corn. On one occasion the miller thought that he would instruct the youth, and teach him to put a bushel of corn in the part of the bag in which he put the stone, and thereby save time and trouble in attending mill. The youth had no idea of making innovations by adopting "new" plans, or of disregarding the teachings and example of his father, and replied: "I will not do so; dad always went to mill that way, and what he did was good enough for me." No evidence is left on record that the young man ever changed his "system" of going to mill! nor is he the only one that ever lived, or yet lives, who has been unwilling to change his opinions, plans of operation, "systems," etc. History—civil, political, moral and ecclesiastical—is replete with such instances. Let us, for illustration, refer to some.

When Copernicus, Galileo and Kepler advanced their hypotheses about the revolutions of the planetary system, but few astronomers were disposed to believe them. Copernicus was afraid of—dreaded—opposition! Galileo was imprisoned. A man was regarded as a heretic in science and religion who believed that the earth moved! Persons are now living who were taught that there were but *four* elements—fire, air, earth and water, and any one who dared to gainsay the teaching would have been considered visionary—had "optics keen to see what was not to be seen." I have no doubt that when Harvey discovered the circulation of the blood, and demonstrated and explained it to Charles I, King of England, many physicians in the great metropolis of the English government, who believed in the "old" teachings and "systems" of physiology, were amused, and perhaps laughing about the "new" notions of the "new" dreamer!

The history of every article used as a medicine, and every remedy resorted to as a means of curing diseases or accidents teaches, in language unmistakable, and loud as thunder, that *it* was at *first* a "new" remedy! Let the physician who still contends that "calomel, the lancet, rhubarb, jalap, opium and tartar emetic" are sufficient to cure *all* "the diseases to which human nature is heir," refer to the history of each article, and, perhaps, he too will learn that they were, when first used, regarded by many as not only "new" but very "dangerous" remedies! As *young* as I am, I remember when one or two grains of quinine were regarded as proper quantities to be given at a dose, and when I commenced the practice of medicine in a miasmatic section, in which I had to contend with remittent, intermittent and congestive fevers, and, according to the teaching and practice of Professors Ford and Dugas, of the Medical College of Georgia, administered quinine in doses of five, ten, fifteen, twenty, thirty or forty grains, according to the emergency of the case, the "*older*" members of the profession in that section considered me "a dangerous young doctor," and an estimable and intelligent old gentleman replied to me in a meeting of a medical society, that he would not permit me to practice in his family if I gave such large doses of quinine! But notwithstanding such opposition, I "went on my way rejoicing" at the success attending the practice, especially as I knew that those profound thinkers and practitioners, Ford and Dugas, had taught and successfully adopted the practice, whilst the older gentlemen kept their patients in bed for four and six weeks with "small doses of calomel, ipecac and nitrate of potash," and, at times, during the intermission and remissions of intermittent and remittent fevers, they would give every two or three hours one or two grains of quinine!

About twenty-five years ago, Dr. Norwood, of South Carolina, revived the use of an *old* medicine—one that had been considered too dangerous to use, and, consequently, had been "consigned to the tomb of the Capulets." But he, with undaunted spirit, cried aloud in its behalf, until it is *now* considered an *old* remedy. So will it be with bromide of potash, hydrate of chloral, and croton-chloral hydrate, to say nothing about anæsthetics, which were introduced into general practice within the last twenty-five years!

But I desire to call the attention of those of the medical profession who are opposed to the use of "*new* remedies" to some prescriptions which are older than some of the old remedies now in use!

Professor L. S. Joynes, of the Medical College of Virginia, has referred to some "old remedies" which, perhaps, our old friends had better use, as they might be less dangerous and more palatable than "calomel and jalap!"

Dr. Joynes has obtained a copy of the Paris *Pharmacopœia* of 1758, and in an article published in the *Virginia Medical Monthly*, May, 1874, entitled "Curiosities of Medical History," he mentions a number of *old* remedies, used in the city of medical learning, and of fine arts, fashions, refinement and good taste. I would very respectfully call the attention of all who are opposed to "new remedies" to that article, with the request that they read and reflect upon the same.

As many may not have the opportunity of seeing the article of Dr. Joynes, I will mention some of the medicines, published as "official" in the Paris *Pharmacopœia* of 1758:

"Oil of bricks," oil of young puppies, oil of earth-worms, and pigeon's dung, of which was prepared a plaster styled *emplastrum diabotanum.* The turtle (or terrapin,) both of land and water, the lizard, the frog, the toad, the viper, the earthworm, the snail, the wood-louse or millipede, the ant, the bee, the crab, the crawfish, the scorpion, the black or dung-beetle ("or tumble-bug.") Great "medicinal virtues were ascribed to each of these ignoble animals." There was prepared a "restorative syrup of turtles," a soothing preparation styled *balsamum tranquillans*, for which five toads are directed to be boiled alive, with other ingredients. To make a famous plaster (*emplastrum de ranis*,) "the formulæ calls for twenty-four live bull frogs, a pound of earth-worms, oil of frogs, powder of viper, and other choice ingredients." The viper appears to have occupied a high position in medical estimation. There were the powder of vipers, a broth of vipers, or viper tea, jelly of vipers, syrup of vipers, troches or lozenges of vipers, a volatile spirit, and a volatile oil of vipers, besides being an ingredient of quite a number of compound preparations, it was prescribed in no less than *fourteen different officinal forms.* Then they prescribed the syrup and broth of turtles, sow-bugs, bees, snails, and syrup and broths of all kinds of bugs and animals! Ivory, the wild boar's tusk, the jawbone of the pike, hare's fur, crab's eyes and claws, raw silk, the spawn of the frog, the blood of the wild goat, the excrement of the dog, the cow, the peacock, the common fowl, the goose and the pigeon were all used. As Dr. Joynes says, "a charming assortment for the druggists to keep in store and manipulate."

Altogether, almost the whole materia medica was one grand *hum*-bug!

The Chinese practice of the present day is about as good as that of Paris in 1758! The Chinese must have obtained a copy of the Paris *Pharmacopœia*, for their materia medica is composed of pretty much the same articles. And yet a short time since a Chinese physician advertised for practice in the city of New York! I have read the system of Chinese practice, and find that *snakes*, and snakes' skins enter largely into the composition of remedies for nearly every disease!

I also refer "old fogies," for their earnest consideration, to the following remedies, which were wont to be prescribed by the illustrious Sydenham.

For intermittent fevers of the years 1661, 1662, 1664, he prescribes thus:

"Take of the conserve flowers of borage and bugloss, each an ounce; conserve of rosemary, half an ounce; candied citron-peel and nutmeg, and Venice treacle, of each three drachms; confections of alhermes, two drachms; mix them up into an electuary, of which let him take the quantity of an hazel nut, morning and night; drinking after it six spoonfuls of the following juice:

"Take of the distilled water of meadow sweet, and treacle water, of each three ounces; syrup of cloves, an ounce; mix them together."

"On the intermediate days, during the course of the cure, give them the following electuary, or some other medicines of the like kind:

"Take of the conserve of Roman wormwood, of rosemary, and of Venice treacle, each an ounce; of the conserve of orange-peel, of candied angelica and nutmeg, each half an ounce; syrup of cloves, enough to make the whole into an electuary, of which let the quantity of a nutmeg be taken twice a day, drinking after each dose a small draught of canary, wherein cowslip flowers have infused cold."

In the treatment of bilious colic, he "commonly" used the following:

"Take of the roots of madder and tumeric, each an ounce; of the roots, together with the leaves of the greater celadine, and the tops of the lesser centuary, each a handful; boil them in equal quantities of Rhenish wine, and spring water to a quart, to which, when strained off, add two ounces of the syrup of the five opening roots; mix them together for an apozem, of which let the patient take half a pint warm, every morning and night, till the cure be completed."

And thus would he "physic" one afflicted of the pleurisy:

"Take seven blanched sweet almonds, the seed of melons and pumpkins, of each half an ounce; the seeds of white poppies, two drachms; beat them together in a marble mortar; then pour on by degrees a pint and a half of barley water; mix well, and, when strained, add two drachms of rose water, and half an ounce of white sugar. Let four ounces be taken every fourth hour."

The following simple emulsion and electuary were for treatment of "the rheumatism:"

"Take of distilled waters of lettuce, purslain and water lily, each four ounces; syrup of lemons, an ounce and a half; syrup of nolets, an ounce; mix them together for a ju e), of which let the patient drink at pleasure, or of the emulsion above set down in the cure of pleurisy."

Again: "Take of the conserve of garden scurvy grass, two ounces; conserve of wood sorrel, an ounce; compound powder wake robin, six drachms; syrup of oranges, enough of the whole into an electuary, two drachms of which is to be taken three times a day, for a month, drinking after it three ounces of the following distilled water:

"Take of garden scurvy grass, eight handfuls; of water cresses, brook lime, sage, and mint, four handfuls; the peels of six oranges; nutmegs bruised, half an ounce. Infuse them into six quarts of mum, and draw off three quarts for use in a common still."

Then for quinsy this:

"Take of the distilled waters of plantain, red roses, and frog spawn, of each three ounces; three whites of eggs, beat to a liquor, white sugar, three drachms; mix them together for a gargism."

And again, we are modestly informed by our *Patriarcha tribarius Æsculapii* that, "for the benefit of young physicians, he will communicate" the following treatment for the gout:

"Take of the roots of angelica, sweet flag, masterwort, elecampane, the leaves of worm-wood, the lesser centuary, white hore-hound, germander, ground pine, scordium, common calamint, feverfew, wild saxifrage, St. John's wort, golden rod, thyme, mint, sage, rue, holy thistle, pennyroyal, southern wood, the flowers of camomile, tansy, lily of the valley, English saffron, the seeds of treacle, mustard, garden scurvy grass, caraway and juniper berries, of each a sufficient quantity. Let the herbs, flowers and roots be gathered when they are in the utmost perfection; dry them in paper bags until they are reducible into a fine powder. Take six o 'nces of each, well mixed together, add enough of clarified honey and canary to make the whole into an electuary, of which let the patient take two drachms morning and night."

I copy the above prescriptions from Sydenham's Practice, now in my possession. Although an advocate for all *good* new remedies, I would not agree to discard any of the *old* remedies, which the experience of years has proven to be valuable—not even *blood-letting!* I still have and use "calomel, ipecac, rhubarb, jalap, tartar emetic, lobelia, opium and the lancet," but cannot reject, with the lights before me, anæsthetics, hydrate of chloral, croton-chloral, nit a e of amyl, the hypodermic syringe, and many other "new" remedies too numerous to mention.

